# Latest Advances in Endothelial Progenitor Cell-Derived Extracellular Vesicles Translation to the Clinic

**DOI:** 10.3389/fcvm.2021.734562

**Published:** 2021-10-04

**Authors:** Amankeldi A. Salybekov, Aidyn D. Kunikeyev, Shuzo Kobayashi, Takayuki Asahara

**Affiliations:** ^1^Division of Regenerative Medicine, Department of Center for Clinical and Translational Science, Shonan Kamakura General Hospital, Kamakura, Japan; ^2^Shonan Research Institute of Innovative Medicine, Shonan Kamakura General Hospital, Kamakura, Japan; ^3^Kidney Disease and Transplant Center, Shonan Kamakura General Hospital, Kamakura, Japan; ^4^Department of Software Engineering, Kazakh National Technical University After K.I. Satpayev, Almaty, Kazakhstan

**Keywords:** extracellular vesicles, endothelial progenitor cells, exosomes, miR (microRNA), clinical application of EPC exosomes

## Abstract

Almost all nucleated cells secrete extracellular vesicles (EVs) that are heterogeneous spheroid patterned or round shape particles ranging from 30 to 200 nm in size. Recent preclinical and clinical studies have shown that endothelial progenitor cell-derived EVs (EPC-EVs) have a beneficial therapeutic effect in various diseases, including cardiovascular diseases and kidney, and lung disorders. Moreover, some animal studies have shown that EPC-EVs selectively accumulate at the injury site with a specific mechanism of binding along with angiogenic and restorative effects that are superior to those of their ancestors. This review article highlights current advances in the biogenesis, delivery route, and long-term storage methods of EPC-EVs and their favorable effects such as anti-inflammatory, angiogenic, and tissue protection in various diseases. Finally, we review the possibility of therapeutic application of EPC-EVs in the clinic.

## Introduction

Endothelial progenitor cells (EPCs) have been widely used to treat cardiovascular ischemic diseases since their discovery in 1997 (1). Initial clinical trials, in parallel with preclinical studies, raised hopes of cures for life-threatening ischemic diseases (2). In subsequent studies, EPC biology was further investigated and it was found that after long-term culture of between 15 and 21 days, cobblestone-shaped colonies emerged, called blood endothelial outgrowth cells (3). The phenotypes of these cells are similar to those of the adult endothelial cells and have a greater proliferative rate (3); Yoder‘s group (4) found similar cells from umbilical cord blood cells (4). Clinical studies have demonstrated that the origin of EPC is bone marrow, and considering pathological triggers, these cells migrate to damaged tissues and physically contribute to facilitating vasculature (3, 5–7). However, several groups are concerned about the existence of EPCs based on mouse data (8). In addition, the culture of EPCs in diverse systems, different methodologies, and various “misleading terms” has led to confusion in EPC biology and application. To this end, a recent consensus attempted to standardize EPC nomenclature based on cellular phenotypes and biological functions (9). A consensus statement on EPC nomenclature and culture standardization may facilitate progress toward the use of EPC-derived extracellular vesicle (EPC-EV) therapy. Depending on the sequence of appearance in culture, Hur et al. (10) reported two types of EPCs. The first were termed early EPCs or myeloid angiogenic cells that were positive for CD45, CD14, and CD31 markers, and mainly worked via paracrine mechanisms, such as growth factors and EVs (10, 11). The second cell population, named late-EPC or endothelial colony-forming cells (ECFCs), usually appeared in culture at 2 to 3 weeks after cell culturing, and had similar phenotypes as endothelial cells, and enhanced neovascularization in ischemic tissues (10, 12). Recent studies have demonstrated that ECFCs secrete EVs that are crucial for organ restoration (13–15).

**Figure 1:**
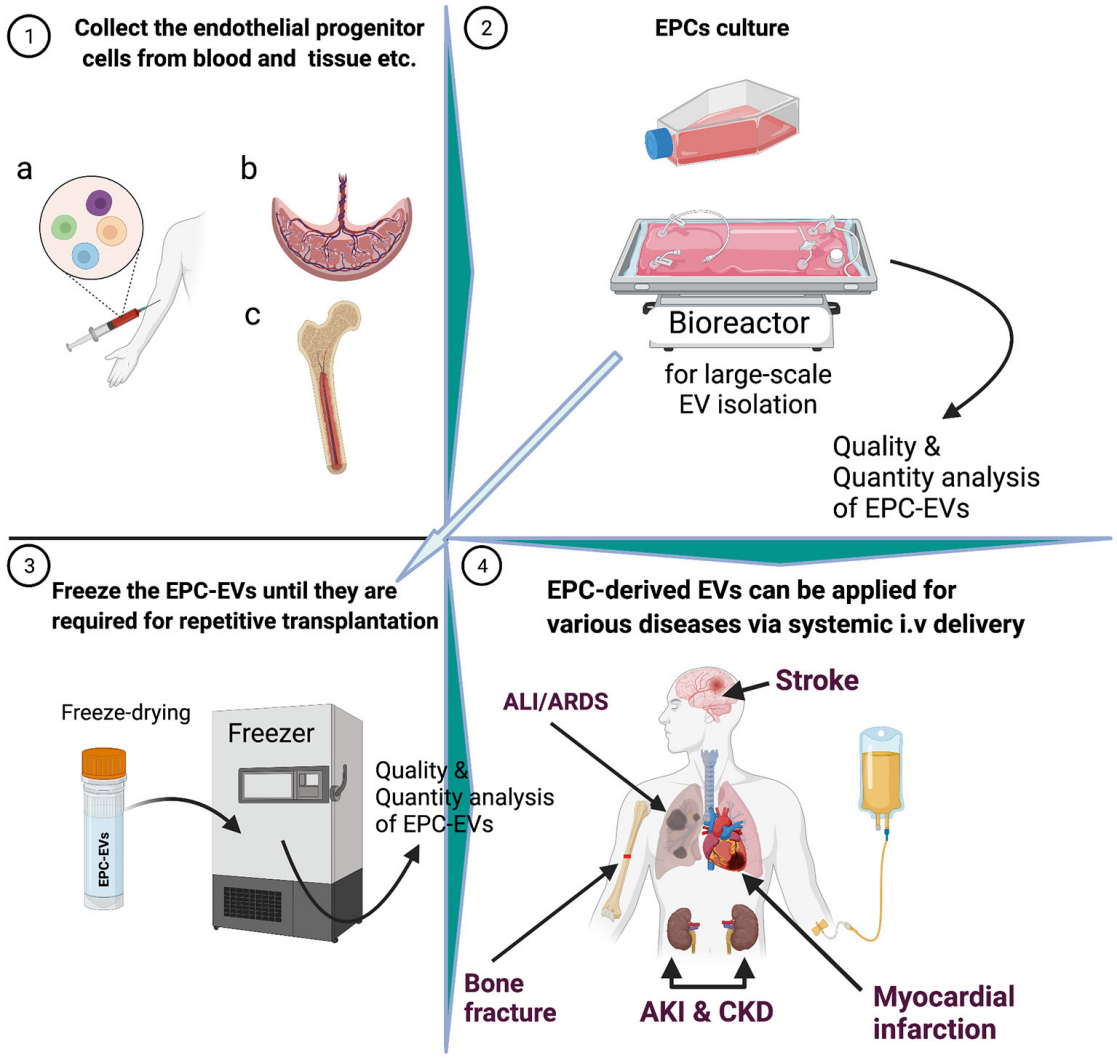
Graphical abstract.

Almost all nucleated cells secrete extracellular vesicles cargo which deliveries nucleic acid and proteins to the recipient cells. The International Society for Extracellular Vesicles consensus recommendation on nomenclature endorses to use “extracellular vesicles” as a generic term for a lipid bilayer particle released from the cell and cannot replicate. Moreover, it has a broader meaning which can cover subtypes like exosomes and microvesicles as well. Depending on the physical size range, EVs divide small (<100 nm), medium (<200 nm), or large (>200 nm), and usually express CD63+, CD81+, Annexin A5, etc., surface markers (16). The small EVs are generated within endosomes as intraluminal vesicles and this complex EVs biogenesis occurs by endosomal sorting complex required for transport (ESCRT) sorting machineries involvement (17). Whereas, medium or large EVs originate by an outward budding at the plasma membrane (Figure 1) (18). There are several methods to isolate secreted EVs such as classical differential centrifugation, density gradient centrifugation, size-exclusion chromatography, ultrafiltration, immunocapture, precipitation, and tangential flow filtration, etc. (Figure 1) (16). Each EV isolation strategy or its working principle along with their advantages and disadvantages was reviewed previously (19).

**Figure 2:**
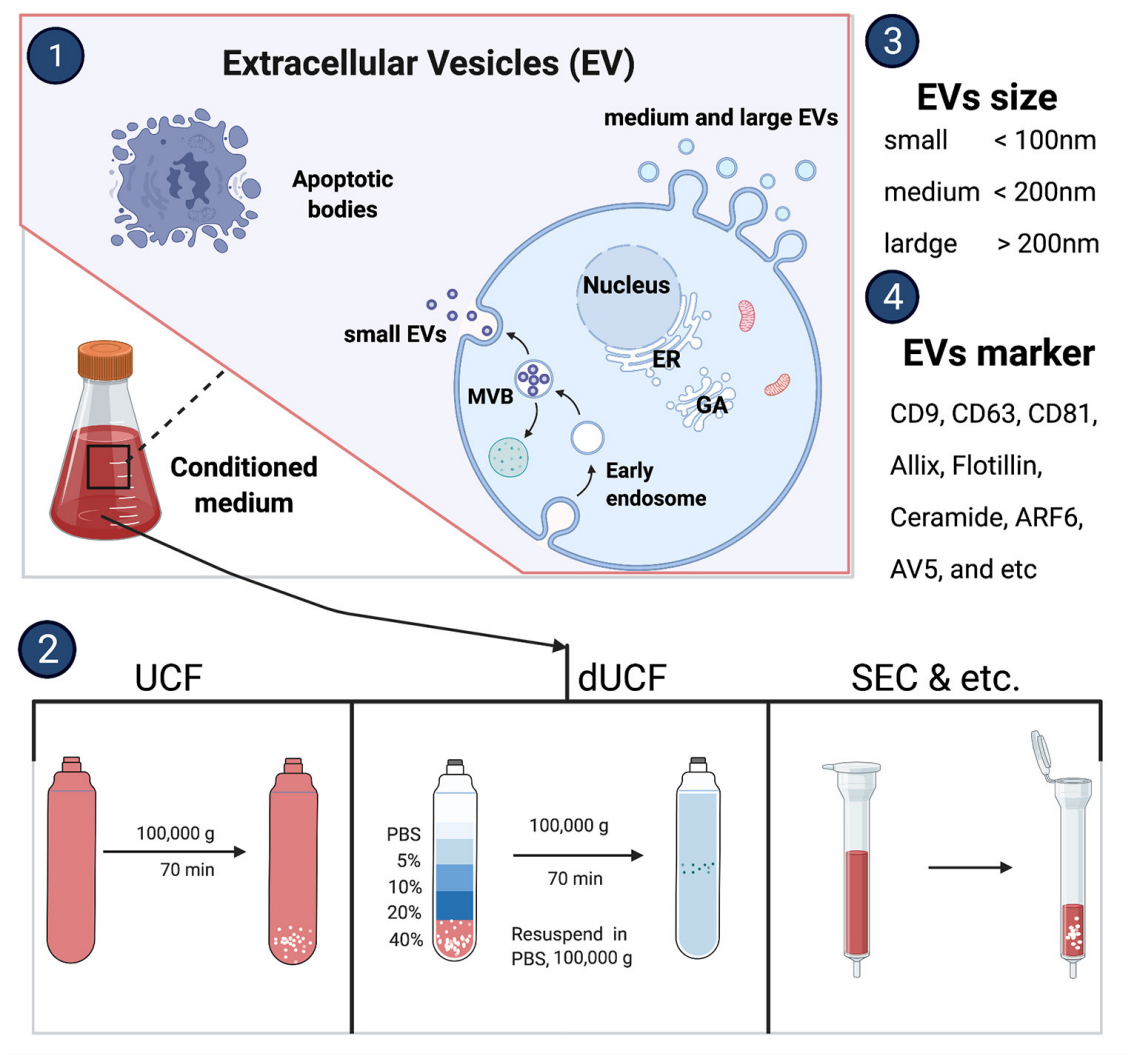
Extracellular vesicles biogenesis and isolation methods. UCF, ultracentrifuge; dUCF, density gradient centrifugation, size-exclusion chromatography.

A seminal study showed that EPCs secrete microvesicles, and the latter activate an angiogenic program in endothelial cells via horizontal transfer of mRNA (20). Subsequently, preclinical studies showed that EPC-EVs have superior therapeutic effects on various ischemic diseases (Figure 2) (21–23). In the last decade, numerous studies on EPC-EVs have shed light on EV biogenesis, uptake, and mechanism of action (24–27). This review highlights recent advances in the biogenesis, biological functions, route of delivery, and long-term storage of EPC-EVs. Finally, we describe potential translation to the clinic and regions of application in the context of various ischemic and inflammatory diseases.

## EPCs vs. EPC-EVs

**Figure 3:**
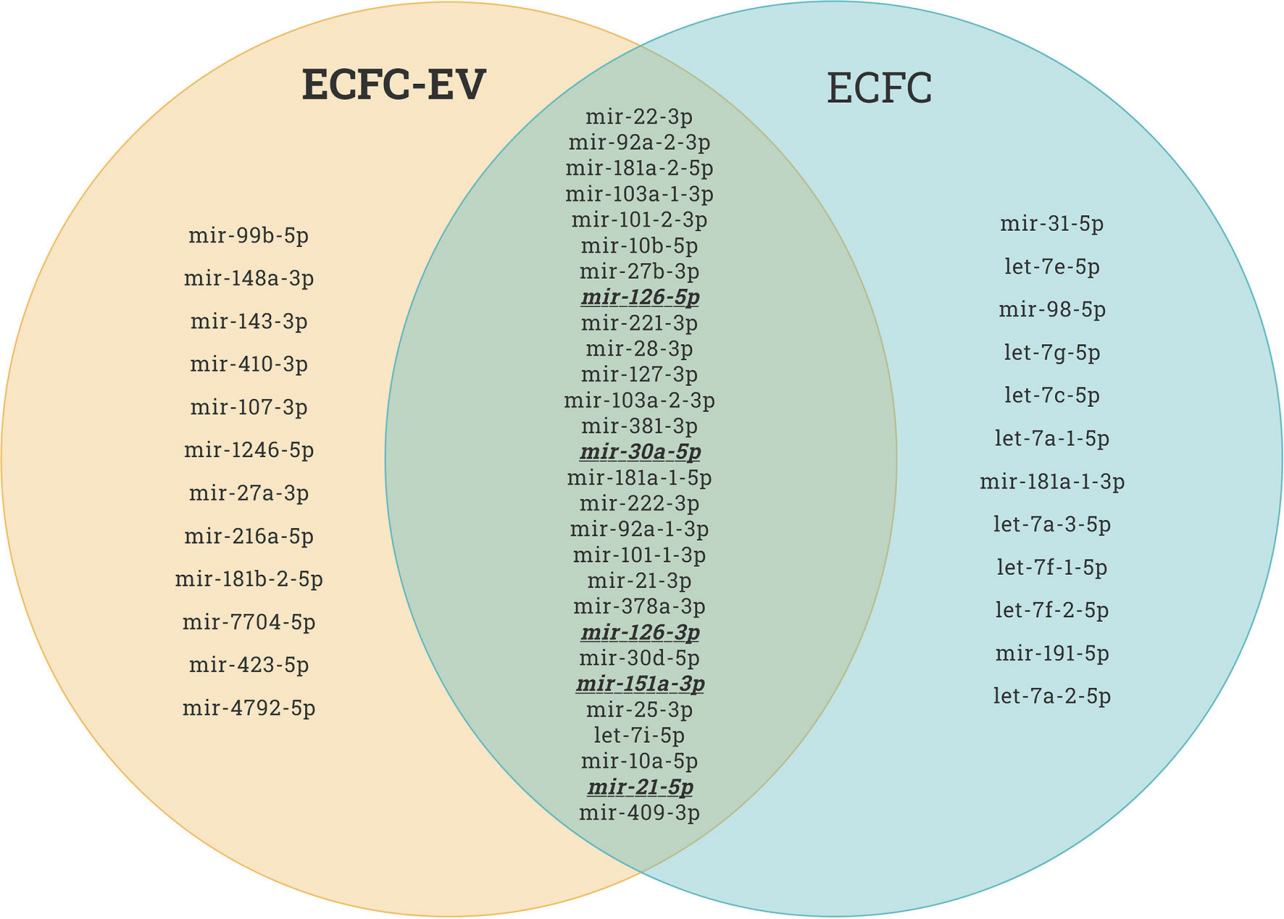
Endothelial colony forming cell (ECFC)-derived microRNAs (miRs) vs. ECFC-extracellular vesicle (EV)-derived miRs. As shown in the Venn diagram, the majority of the top upregulated human miRs of both ECFC-derived and ECFC-EVs-derived miRs comprise similar miRs. This provides evidence that EPCs work via paracrine factors in organ regeneration. This miR sequence data was generated from PMID: 28631889.

**Figure 4:**
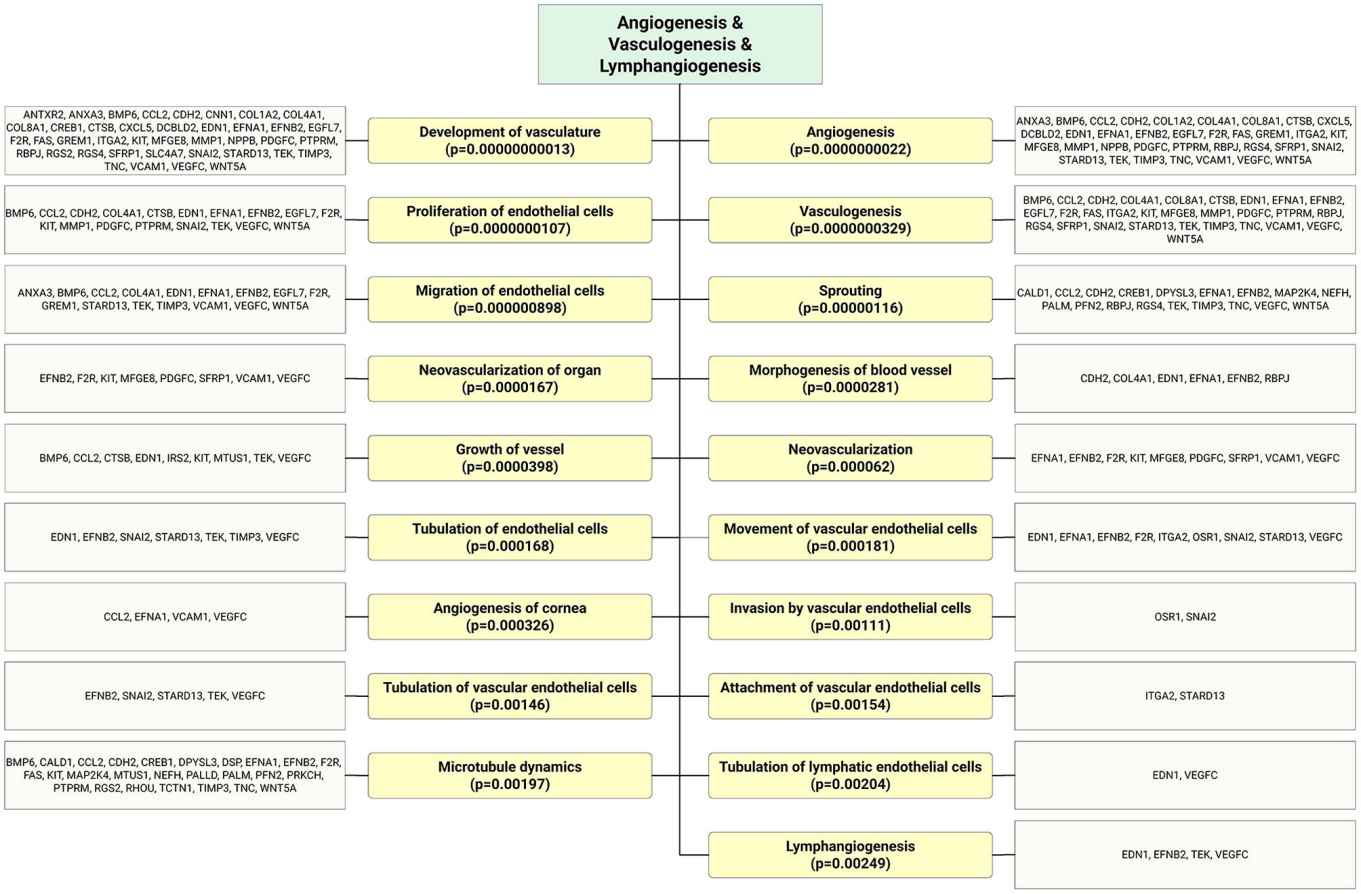
As shown in the flow chart that highly expressed ECFC-EC-derived miRs biological targets were predicted *in silico*. The most significantly enriched functional annotation were Angiogenesis, vasculogenesis, and lymphangiogenesis. This miR sequence data was generated from PMID: 28631889. MicroRNA target genes were predicted using IPA with information from TargetScan, miRecords, and TarBase databases.

Recently, it has been reported that therapeutic cell transplantation-related effects for cardiovascular diseases are a result of paracrine mechanisms and not from direct cell contribution to damaged organs (28). Regardless of the target delivery technique, the long-term engraftment of cells is limited; hence, the striking short-term improvement in ischemic organ function after cell transplantation is mainly associated with paracrine trophic factors such as EVs (29). It has been shown that early EPC populations are contaminated with hematopoietic cell subsets, such as monocytes (30), and the latter secrete various nanoparticles. In contrast, late EPCs have specific phenotypes and biological functions similar to endothelial cells, and secrete angiomiRs-shuttled EVs, which are a key genetic material for neovascularization of ischemic tissues (14, 31, 32). Dozens of preclinical studies have demonstrated EPC-EV effects that are superior to those of the ancestor (33). EVs possess numerous advantages over cell-based therapies in the context of regenerative medicine in terms of (1) cargo delivery of various favorable miRs responsible for angiogenesis, fibrosis, and cell proliferation; (2) potential for “off the shelf” availability and respective for repetitive transplantation; (3) cell-free biological products that may be utilized as drug carrier systems in the pharmaceutical industry, and finally (4) generally reduced immunogenicity owing to which allogenic transplantation is an additional benefit. The abovementioned benefits are crucial for treating either acute or chronic diseases. The latter listed major advantages of the EVs are linked to less immunogenic than their parental cells because of the lower abundance of transmembrane proteins such as MHC complexes on their surface (34). Unlike live cells, EVs have a long shelf life and may be transported and stored for long periods (see the section on long-term preservation and storage of EVs). In the representative Venn diagram (Figure 3) (transcriptome data from previous publication PMID: 28631889), we summarize the similarities and differences between ECFC-derived microRNAs (miRs) and ECFC-EV-derived miRs (35). It can be clearly seen that the majority of parent cell-derived miRs (ECFC-miRs) can be found in ECFC-EV-derived miRs, suggesting a similar transcriptome profile along with the mechanism of action (Figure 3). A previous study showed that the therapeutic potential of EPC-EVs is superior in terms of enhancing neovascularization and recovery in a murine hind limb ischemia model (12). The mechanism of activation of the angiogenic program in quiescent endothelial cells is linked by horizontal transfer of genetic materials such as angiomiR, RNA, and proteins (12, 13). Of note, ischemia itself is a trigger for angiogenesis. However, angiogenesis-qualified angiomiRs accelerate not only angiogenesis but also proliferative and anti-apoptotic effects (Figure 4). Collectively, well-packed EPC-EVs have a great advantage in preserving ischemic tissue from injury, and future studies are warranted to define the beneficial effects of EPC-EVs.

## EPC-EVs in Cardiovascular Diseases

**Figure 5:**
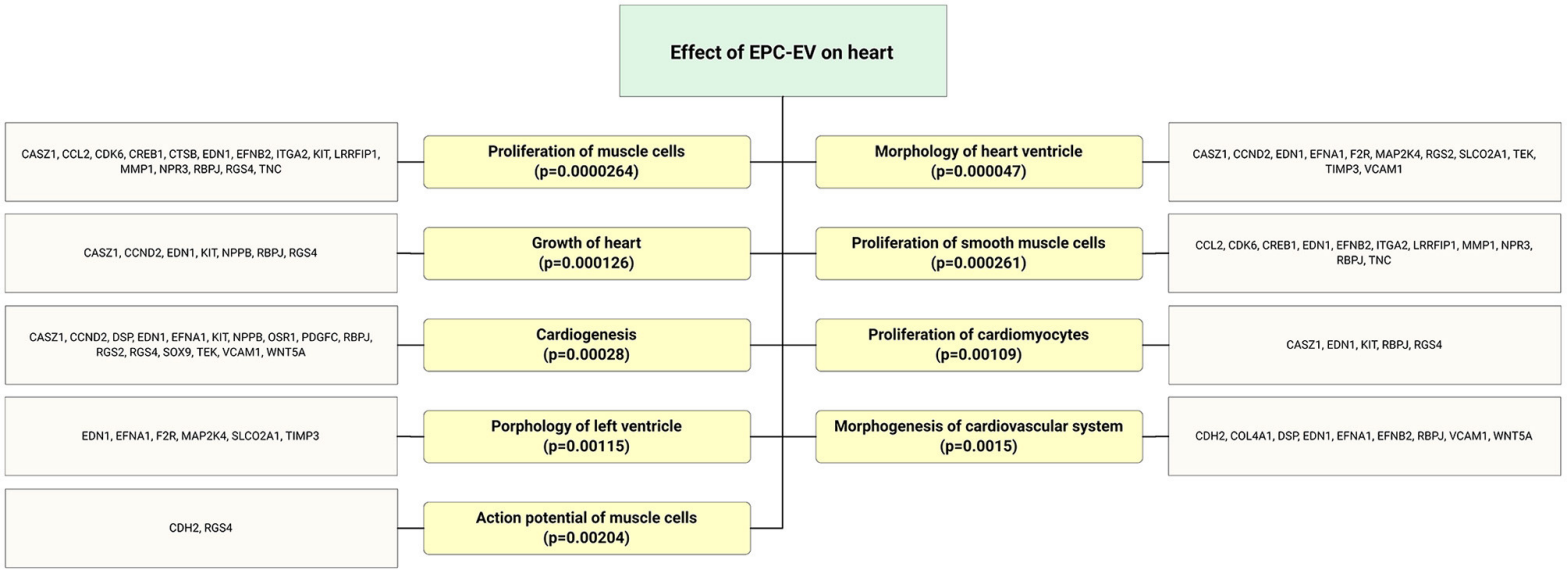
Flow chart depicts highly expressed ECFC-EC-derived miRs biological targets that were predicted *in silico*. The significantly enriched functional annotation was associated with heart tissue repair. This miR sequence data was generated from PMID: 28631889. MicroRNA target genes were predicted using IPA with information from TargetScan, miRecords, and TarBase databases.

Cardiovascular diseases are the leading cause of mortality and morbidity in the globe (36). It has been shown that therapeutic neoangiogenesis with EPCs is a promising strategy for treating advanced cardiovascular diseases and preventing major adverse events (37). Similar transcriptome profiles of EPC-derived EVs to the EPCs facilitate therapeutic application EPC-EVs in CVD. Yue et al. (38) demonstrated that EPC-derived exosome treatment enhanced left ventricle cardiac function, reduced cell apoptosis, diminished myocardial scar size, and promoted post-myocardial infarction neovascularization. Previous studies have shown that sonic hedgehog modified progenitor cells (CD34+) actively secrete exosome cargo and carry various reparative molecules to cure the ischemic myocardium (39, 40). EPC-EVs regulate cardioprotection by orchestrating cell angiogenesis, migration and adhesion, cell proliferation, and cell differentiation processes (Figure 5). Target gene expression analysis of EPC-EV-derived miR revealed that heart regeneration and protection enriched functional gene upregulation (Figure 5). Cardioprotective properties of EPC-derived EV is associated with miR-218-5p and miR-363-3p overexpression. The latter facilitated cardiac function via enhanced neoangiogenesis and inhibited myocardial fibrosis (41). Moreover, EPC-EVs treatment promoted mesenchymal-endothelial transition and along with protective effect to myocardial infarcted tissues (42). Recently, Chen et al. (33) showed that using EPC-EVs and encapsulation with a hydrogel could increase biological activity for up to 3 weeks through sustained release. Furthermore, the injected hydrogel system for sustained EPC-EV delivery into the ischemic myocardium augmented hemodynamics via increased vessel density in the peri-infarcted area along with reduction in myocardial scar formation. Interestingly, the regenerative efficacy of hydrogen-encapsulated EPC-EVs is not inferior to that of the parent cells or EPCs (33). Repetitive systemic transplantation of EVs is a simple delivery option. We have recently shown that systemic repetitive transplantation of EVs derived from regeneration-associated cells in a rat model of myocardial IR injury significantly enhanced cardiac functions, such as ejection fraction, and preserved mitral regurgitation. In addition, we could not observe anti-donor immune responses even when EV transplantation was performed in allogeneic settings.

Taken together, EPC-EVs have anti-inflammatory and anti-fibrotic properties and may enhance angiogenesis in the ischemic myocardium.

## EPC-EVs in Acute Lung Injury and Acute Respiratory Distress Syndrome (ALI/ARDS) Pathology

Systematic reviews have demonstrated that the mortality rate in ALI/ARDS is between 36 and 44% and is usually induced by various etiologies such as sepsis, pneumonia, and severe traumas (43). The high mortality rate in ALI/ARDS facilitates various “cell-free” therapeutic EVs, including EPC-EVs. Emerging data shows that EPC-EV administration markedly reduced lipopolysaccharide-induced lung inflammation compared to that in the control groups, indicating a strong anti-inflammatory effect of EVs. Histological examination of the EPC-EV-administered group showed limited alveolar edema and lung neutrophil infiltration, and reduced cytokine/chemokine levels in the bronchoalveolar lavage fluid (44). Mechanistically, EPC-EV contains abundant miRNA-126, and overexpression of miRNA-126-3p can target phosphoinositide-3-kinase regulatory subunit 2, whereas overexpression of miRNA-126-5p inhibits the inflammatory alarmin high mobility group box 1 (HMGB1) and the permeability factor vascular endothelial growth factor (VEGF) α (44). Wu et al. (45) reported different mechanistic insights into EPC exosome-mediated transfer of miR-126 to endothelial cells such as the selective expression of SPRED1 and the enhancement of RAF/ERK signaling pathways that were primarily responsible for restoring the acute-injured lung. In summary, EPC-EVs have a beneficial effect in improving ALI/ARDS outcomes, and further studies are necessary to define optimal and targeted EV delivery methods to the site of injury.

## EPC-EVs in Sepsis

Sepsis is a systemic inflammation induced mainly by microorganisms, leading to organ dysfunction. Recent studies have highlighted that EPC transplantation has a beneficial effect on animal models of sepsis (46, 47). Mechanistically, various pro-inflammatory cytokines induced by pathogen-associated molecular patterns (PAMPs) and damage-associated molecular patterns (DAMPs) in peripheral blood cause vascular injury and increase permeability (47). Consequently, in response to vascular injury, EPCs mobilize in an SDF1a-dependent manner and directly recruit to the injury site and differentiate into mature endothelial cells (47, 48). Fan et al. (46) demonstrated that EPCs and SDF1a administration synergistically improves survival in septic animals via enhanced miR-126 and miR-125b expression, which is believed to play key roles in the maintenance of endothelial cell function and inflammation. Later, they demonstrated that the protective effect of EPCs on the microvasculature after sepsis occurs via exosome-mediated transfer of miRs such as miR-126-3p and 5p (49). EPC-EVs miR-126-5p and 3p suppressed DAMP-induced *HMGB1* and vascular cell adhesion molecule 1 (VCAM1) levels, whereas inhibition of miR-126-5p and 3p through transfection with miR-126-5p and 3p inhibitors disrupted the beneficial effect of EPC exosomes. Thus, EPC-EVs prevent adverse septic complications via miR-126 delivery (49).

## EPC-EVs in Acute Kidney Diseases

Ischemia/reperfusion is a major cause of acute kidney injury (AKI) in humans, and is associated with tubular cell necrosis and endothelial cell dysfunction or loss. Growing evidence has shown that the therapeutic potential of EPC-EVs is superior in terms of acute kidney disease. Vinas et al. (15) used ECFC-derived EVs in an acute kidney injury mouse model and showed that miR-486-5p enriched ECFC exosomes significantly reduced ischemia-induced kidney injury. Histologically, exosome treatment decreased the infiltration of neutrophils along with diminished apoptosis and caspase-3 activation. Moreover, administration of exosomes to acute kidney injury-induced animals caused potent protection against kidney injury after 24 h, as evidenced by normalization of plasma creatinine and blood urea nitrogen to the same level as that in the healthy control. Mechanistically, miR-486-5p enriched ECFC exosomes target to reduce the phosphatase and tensin homolog, and stimulate the Akt phosphorylation pathway for ischemic tissue preservation (15). Cantaluppi et al. (13) demonstrated that EPC-EVs carrying miR-126 and miR-296 protect against experimental acute renal IRI, as evidenced by a significant decrease in serum creatinine and blood urea nitrogen levels and improvement in histological signs of microvascular and tubular injury. It is well-known that EPC-EVs exert miR-126 and have strong angiogenic and anti-apoptotic potential (23). In another study, EPC-EV transplantation rescued an experimental model of anti-Thy1.1-induced glomerulonephritis via inhibition of antibody- and complement-mediated injury of mesangial cells (50). In a review article, Sun et al. (51) summarized that stem/progenitor cell-derived EVs, including EPC-EVs, have beneficial effects such as anti-inflammatory, anti-apoptotic, anti-fibrotic, and may also promote renal cancer progression. In summary, EPC-EVs were shown to have a strong renoprotective effect in an acute kidney injury model, and future studies are warranted to extend their application to chronic kidney diseases.

## EPC-EVs in Bone and Connective Tissue Repair

**Figure 6:**
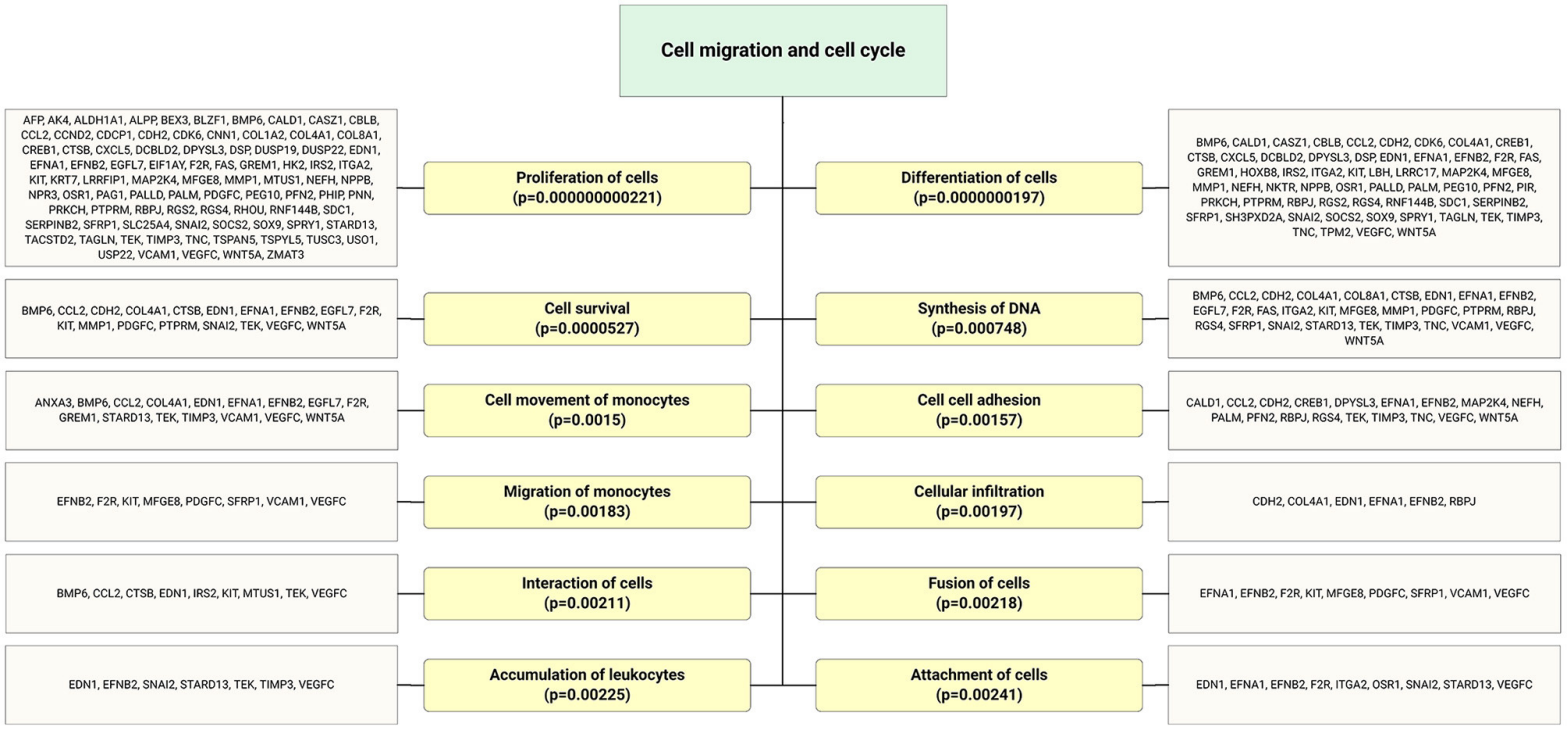
Highly expressed ECFC-EC-derived miRs biological targets were predicted, and the most significantly enriched functional annotations were cell migration and cell cycle. This miR sequence data was generated from PMID: 28631889. MicroRNA target genes were predicted using IPA with information from TargetScan, miRecords, and TarBase databases.

**Figure 7:**
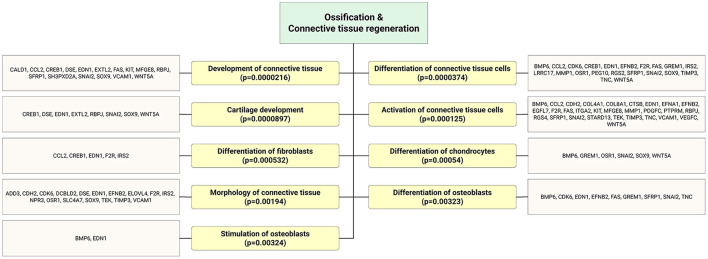
Bioinformatics analysis showed that significantly enriched functional annotation related to bone and connective tissue regeneration. This miR sequence data was generated from PMID: 28631889. MicroRNA target genes were predicted using IPA with information from TargetScan, miRecords, and TarBase databases.

Accumulating evidence demonstrates that EPCs have beneficial effects on bone regeneration by secreting trophic and paracrine factors (52, 53). Pang et al. (54) showed that EPCs modulate the survival, migration, and differentiation potential of osteoclast precursors through the VEGFR-2, CXCR4, Smad2/3, Akt, ERK1, and p38 MAPK pathways (Figure 6). Interestingly, target genes of highly expressed EPC-EV miRs yielded several significant bone and osteoblast differentiation-enriched functional categories (Figure 7). Through *in silico* experiments, Qin et al. (55) showed that EPC-EVs regulate the osteoblastic differentiation of bone marrow-derived mesenchymal stromal cells by inhibiting the expression of osteogenic genes and increasing proliferation. This suggests that EPC-EVs are able to control osteogenesis and have beneficial effects on connective tissue development, such as fibroblasts and chondrocytes (Figure 7). A preclinical study showed that EPC-EVs have a strong therapeutic effect on distraction osteogenesis by stimulating angiogenesis and osteogenesis (56). The aforementioned therapeutic advantage of EPC-EVs in bone and connective tissue regeneration expands its application to cure various skeletal muscle diseases.

## Angiogenic Properties of EPC-EVs

Recent studies have shed light on the biological activity and function of EPC-Ev-derived miRs in various *in vitro* and *in vivo* models. Dellet et al. (35) demonstrated the high expression levels of 15 miRs identified in ECFC and ECFC-derived EVs such as miR-10a/b, miR-21-5p, miR-30a-5p, miR-126-5p, let-7 families, and miR151a-3p (Figure 3 and Table 1). We further investigated the angiogenic/vasculogenic properties of EPC-EV-derived miRs *in silico*. As shown in Figure 4, the EPC-EVs-derived miR targets are expressed on angiogenesis- and vasculogenesis-related genes. The majority of the neovasculogenesis phenomenon is coupled with cardiovascular system development and function (Figure 4). Plummder et al. (59) reported that EPC-derived miR-10b and miR-196b overexpression activates VEGF, and the latter enhances breast tumor vasculature. Interestingly, downregulation of miR-10b and miR-196b significantly inhibited tumor angiogenesis in mice, indicating a strong angiogenic potential. miR-126-5p and miR-126-3p overexpression promoted EPC migration and tube-like structure formation in ischemic cardiomyopathy patients *in vitro* (57). Moreover, transplantation of miR-126-3p-overexpressing EPCs into a rat model of MI showed left ventricular hemodynamic functions along with histological improvements (57). Mathiyalagan et al. (23) also demonstrated that silencing miR-126-3p from CD34 cell-derived exosomes abolished their angiogenic activity and beneficial function both *in vitro* and *in vivo*. Furthermore, injection of CD34 cell-derived exosomes increased miR-126-3p levels in mouse ischemic limbs but did not affect the endogenous synthesis of miR-126-3p, indicating a direct transfer of functional miR-126-3p to the ischemic tissue (23).

**Table 1:** EPC-derived angio-miR.

**miRs**	**Mechanism of action**	**Target diseases**	**EVs or Exo origin**	**Ref #**
miR-126-3p	*VEGF-A, IL-3, IL-10, IGF-1, ANG1, ANG2*, and *SPRED1*	Enhanced biological function of EPC from patients with ICM. Knocking down miR-126-3p from EPC abolished their angiogenic activity	EPC-derived EVs	(23, 57)
miR-126-5p	*DLK1*	Prevents atherosclerotic lesion formation via DLK1 suppression	EC	(35, 58)
miR-10b	*VEGF* and *HOX*	Promotes tumor growth via enhanced angiogenesis	Circulating EPC, EC	(35, 59, 60)
let-7b let-7f-2-5p let-7f-1-5p let-7i-5p	Proangiogenic paracrine factors and *IL-10* and *IL-12*	ECFC-derived EVs vastly contain various let-7 miR and modulate ischemia-induced angiogenesis. Tumor-associated macrophages phenotypes were changed upon downregulation	ECFC	(35, 61–64)
miR-486-5p	*PTEN* and Akt pathway	Delivery of ECFC exosomes reduces ischemic kidney injury via transfer of miR-486-5p targeting *PTEN*	ECFC	(15, 35)
miR-296-5p	*HGS, VEGFR2, PDGF-b*, and inhibiting *DLL4* and *Notch1*	Augmented primary human brain microvascular endothelial cells angiogenic property	Angiogenic EC	(65)
miR-150	*c-Myb*	MiR-150 significantly promoted the migration and tube formation ability of EPCs *in vitro* and enhanced EPCs' homing, organization, and resolution ability *in vivo*	EPC	(66)

*EPC, endothelial progenitor cells; Exo, exosomes; EC, endothelial cells; ECFC, endothelial colony-forming cells; ICM, ischemic cardiomyopathy*.

## Delivery Routes of EPC-EVs

### Systemic Infusion vs. Local Injection

Previous cell therapy trials have reported that the efficacy of cell therapy is limited by poor engraftment of cells or that engrafted cells disappear several months after transplantation, suggesting a paracrine-based effect on the tissues. Depending on the disease state and location, EV transplantation routes may differ. Classical intravenous transplantation of EPC-EVs has been widely used in preclinical and clinical studies (12, 67) (Table 2). Several beneficial functions of systemic transplantation are listed, including (i) no requirement for special sophisticated delivery techniques, (ii) the immunomodulatory effect of EPC-EVs, and (iii) option for repetitive transplantation that is advantageous for local delivery. Sometimes, the desired results cannot be obtained after one injection of EVs; consequently, repetitive systemic transplantation via the vein is needed, whereas in several diseases, local transplantation is not allowed for this technique. Recently, Yi et al. (27) reviewed 29 publications on the route of administration in preclinical studies and showed that the intravenous route was selected in ~80% of exosome injections, and the remaining exosome delivery routes were intraperitoneal, oral, or local. For instance, an ongoing phase one clinical trial (NCT04327635) on safety evaluation of intracoronary infusion of EV in patients with acute myocardial infarction performs within 20 min after stent placement or post-dilation (whichever is last) (Table 2). This kind of delivery methodology is widely used in previous/also current cell transplantation trials into stent placement or post-dilation vessels to enhance the treatment of damaged organs. Another completed clinical trial (NCT04134676) primary outcome revealed that stem cell-conditioned media-derived EVs therapeutic potential is promising in terms of chronic ulcer size reduction, edema decrease, and presence of granulation signs (Table 2). These macroscopic findings were reported 2 weeks after local delivery of EVs via gel.

**Table 2:** Clinical trials on therapeutic applications of extracellular vehicles (EVs).

**Study Title**	**Status**	**Location**	**EVs or Exo origin**	**ClinicalTrials.gov identifier**
Antiplatelet therapy effect on extracellular vesicles in acute myocardial infarction (AFFECT EV), Phase 4	Completed	Warsaw, Poland and Amsterdam, Netherland	Extracellular vesicles from endothelial cells, leukocytes, and platelets	NCT02931045
Safety evaluation of intracoronary infusion of extracellular vesicles in patients with AMI, Phase 1	Not yet recruiting	Commercial study of drug called PEP	Unknown	NCT04327635
Safety and efficacy of allogenic mesenchymal stem cells derived exosome on disability of patients with acute ischemic stroke: a randomized, single-blind, placebo-controlled, Phase 1, 2 Trial	Active/recruiting	Tehran, Iran	Allogenic mesenchymal stem cells-derived exosome enriched by miR-124	NCT03384433
Effect of plasma derived exosomes on cutaneous wound healing	Active/recruiting	Kumamoto, Japan	Autologous plasma-derived exosomes	NCT02565264
Therapeutic potential of stem cell conditioned medium on chronic ulcer wounds: pilot study in human, Phase 1	Completed	Banten, Indonesia	Stem cell conditioned media-derived EVs	NCT04134676
Effect of saxagliptin and dapagliflozin on endothelial progenitor cell in patients with type 2 diabetes mellitus	Recruiting	District of Columbia, United States	Exosomes released from kidney podocyte	NCT03660683
Autologous serum-derived EV for venous trophic lesions not responsive to conventional treatments (SER-VES-HEAL)	Recruiting	Turin, Italy	Autologous extracellular vesicles from serum	NCT04652531

In most cases, intravenously transplanted EVs accumulate in the liver, lung, spleen, and kidney (27). For target organ delivery, it has been shown that the local tissue inflammatory environment and activation of receptors and ligands (adhesion molecules) play essential roles in EV uptake. This information is valuable for *in vivo* biodistribution of exosomes and the control of dose and potential side effects.

## Local Sustained Delivery System

To achieve better results, a targeted delivery system with sustained release to damaged organs may be required. Chen et al. (33) demonstrated that the injection of EPC-EVs incorporated with shear-thinning gel into the border zone of myocardial infarction improved the hemodynamic function of the heart. The average steady EPC-EV release from the gel continued for over 21 days. This EV delivery strategy may enhance EV retention by damaged tissue owing to the sustained release and has potential for active use in trophic ulcer treatment.

## Mechanism of Uptake and Action of EPC-EVs

The mechanism of EV internalization into recipient or acceptor cells is crucial in terms of intercellular communication. Several EV internalization mechanisms have been presented previously in the scientific literature, such as direct uptake followed by fusion, phagocytosis, and macropinocytosis by the recipient cell membrane (25). Indirect EV uptake mechanisms are sophisticated and work through other pathways, such as the clathrin-dependent and clathrin-independent pathways and lipid raft-mediated, caveolin-mediated, and cell surface protein-mediated endocytosis (24, 25, 68). In addition, recent reports revealed that tissue microenvironment pH is a crucial factor for EV uptake and secretion (69); for instance, in a rodent myocardial ischemia injury model, MSC-EVs internalization into ischemic cardiomyocytes was enhanced compared to that in the non-ischemic counterparts, indicating a low pH condition as the likely mechanism (69, 70). Another factor that is common for the preferential accumulation of EPCs and hematopoietic cells in ischemic tissue is the SDF-1/CXCR4 system (71–73). Recently, Viñas et al. (14) showed that CXCR4/SDF-1α interaction plays an essential role in EPC-derived exosome uptake in a mouse acute kidney ischemia-reperfusion injury model. Interestingly, EPC-EVs selectively targeted the ischemic kidney tissues. Hence, transplanted EPC-EVs were detected 30 min to 4 h after reperfusion only within the proximal tubules, glomeruli, and endothelial cells. However, this preferential internalization into the ischemic kidney was interrupted when exosomes were pre-incubated with the CXCR4 inhibitor plerixafor, suggesting CXCR4/SDF-1 α-dependent EPC-EV uptake in ischemic tissues. Taken together, EPC-EVs internalize to the target cells of the CXCR4/SDF-1α system under ischemic conditions, similar to EPCs, although other EPC-EV internalization mechanisms are essential for non-ischemic diseases.

## Long-Term Preservation and Storage of EVs

One of the major challenges for the prolonged clinical applicability of EVs is the establishment of proper and reproducible preservation and storage conditions without compromising their therapeutic potential. Several studies have shown that different methods of storage, chemical compounds, and temperature range optimization are crucial before translation to the clinic (74–76). Recently, Wu et al. (77) evaluated the effect of storage temperature by storing EVs at 4 °C, −20 °C, and −80 °C for up to 28 days and comparing them to fresh EVs. In comparison to fresh EVs, 1 month of storage at 4 °C and −20 °C changed the size distribution, decreased the quantity and content, and affected cellular uptake and biodistribution of EVs; however, storage at −80 °C did not show such effects. The authors concluded that storage at 4 °C or −20 °C is suitable for short-term preservation, whereas −80 °C would be preferable for long-term preservation of EVs for therapeutic applications (77). Jin et al. (78) reported that EVs are stable under the conditions of 4 °C (for 24, 72, and 168 h), at room temperature (for 6, 12, 24, and 48 h), and repeated freeze-thaw (from one to five times).

Moreover, the assessment of DNA content and functionality in EVs was stable in a changing environment over repeated freeze-thaw cycles (78). Freeze-drying or lyophilization seems to be the most reliable method for preserving EVs (76). The common stabilizers used in lyophilization are disaccharides such as glucose, lactose, sucrose, and trehalose. A comparative study of EV storage at 4 °C or −80 °C and freeze-drying showed that lyophilization preserves size and enzyme activity which are indicators of EV stability (79). In summary, for long-term EV storage, preferable conditions are deep freezing, such as at −80 °C or below, whereas 4 °C may be acceptable for short-term use. For advanced EV applications, it is preferable to store EV using lyophilization methods to optimize the biological function and therapeutic potential of EVs.

## Future Perspectives and Conclusion

Intensive research on endothelial progenitor cells and translation to the clinic for various cardiovascular ischemia diseases has increased our understanding of their therapeutic mechanisms (e.g., paracrine mechanism-based action) and biological function (80–82). EPC-EVs may be considered as a primary candidate for use against certain ischemic diseases, owing to their strong angiogenic, anti-fibrosis, and immunomodulatory properties (12, 20, 21, 23, 33, 57, 59, 80, 82–86) and safety in clinical settings (Table 2). However, there are hurdles to overcome before EPC-EVs can be applied as therapies such as standardization of classification and nomenclature of EPCs and focusing on the question of which EPCs should be used (9, 87). In addition, depending on the origin, such as tissue-derived or circulating EPCs, EPC-EVs cargo may contain/comprise various genetic materials that could influence the clinical outcome and should be carefully considered before therapy. Another aspect that needs to be addressed is EPC culturing conditions, including the effect of culture media, ischemia preconditioning, and composition of EVs, all of which must be investigated precisely using large animal disease models. The development of optimized and scalable isolation of pure, clinical-grade EPC-EVs for off-the-shelf therapy use will increase their significance. To date, most EV-based studies have used intravenous bolus injection methods, although the choice of the EV delivery route depends on the location of the disease.

Nevertheless, completed and ongoing clinical trials (Table 2), as well as numerous preclinical studies (38, 42, 44, 45, 86, 88), indicate that EPC-EV therapy is feasible and that EVs are safe and well-tolerated.

## Author Contributions

AS and AK contributed to the literature research and data collection, and were involved in the draft of the manuscript. AK contributed to figure generation and bioinformatic analyses. AS, SK, and TA contributed to the coordination and design of the review and writing of the final draft of the manuscript. All authors have read and approved the final manuscript.

## Funding

This research was supported by JSPS KAKENHI funding (grant no. 20K17163 to AS).

## Conflict of Interest

The authors declare that the research was conducted in the absence of any commercial or financial relationships that could be construed as a potential conflict of interest.

## Publisher's Note

All claims expressed in this article are solely those of the authors and do not necessarily represent those of their affiliated organizations, or those of the publisher, the editors and the reviewers. Any product that may be evaluated in this article, or claim that may be made by its manufacturer, is not guaranteed or endorsed by the publisher.
